# Striatal But Not Extrastriatal Dopamine Receptors Are Critical to Dopaminergic Motor Stimulation

**DOI:** 10.3389/fphar.2017.00935

**Published:** 2017-12-21

**Authors:** Yuhan Wang, Fu-Ming Zhou

**Affiliations:** Department of Pharmacology, University of Tennessee College of Medicine, Memphis, TN, United States

**Keywords:** basal ganglia, dopamine receptor supersensitivity and synergy, L-3, 4-dihydroxyphenylalanine (L-dopa), globus pallidus, intracranial microinjection, medium spiny neuron, Parkinson’s disease, substantia nigra

## Abstract

Dopamine (DA) is required for motor function in vertebrate animals including humans. The striatum, a key motor control center, receives a dense DA innervation and express high levels of DA D1 receptors (D1Rs) and D2 receptors (D2Rs). Other brain areas involved in motor function such as the globus pallidus external segment (GPe) and the substantia nigra pars reticulata (SNr) and the motor cortex (MC) also receive DA innervation and express DA receptors. Thus, the relative contribution of the striatal and extrastriatal DA systems to the motor function has been an important question critical for understanding the functional operation of the motor control circuits and also for therapeutic targeting. We have now experimentally addressed this question in the transcription factor Pitx3 null mutant (Pitx3Null) mice that have an autogenic and parkinsonian-like striatal DA denervation and hence supersensitive motor response to DA stimulation. Using DA agonist unilateral microinjection-induced rotation as a reliable readout of motor stimulation, our results show that L-dopa microinjection into the dorsal striatum (DS) induced 5–10 times more rotations than that induced by L-dopa microinjection into GPe and SNr, while L-dopa microinjection into the primary MC induced the least number of rotations. Furthermore, our results show that separate microinjection of the D1R-like agonist SKF81297 and the D2R-like agonist ropinirole into the DS each induced only modest numbers of rotation, whereas concurrent injection of the two agonists triggered more rotations than the sum of the rotations induced by each of these two agonists separately, indicating D1R–D2R synergy. These results suggest that the striatum, not GPe, SNr or MC, is the primary site for D1Rs and D2Rs to synergistically stimulate motor function in L-dopa treatment of Parkinson’s disease (PD). Our results also predict that non-selective, broad spectrum DA agonists activating both D1Rs and D2Rs are more efficacious anti-PD drugs than the current D2R agonists.

## Introduction

The critical motor function of the brain dopamine (DA) system in animals and humans is amply demonstrated by naturally occurring DA neuron loss-induced Parkinson’s disease (PD) (Parkinson, 1817; Kish et al., 1988; Hornykiewicz, 2001), experimental DA depletion-induced akinesia in animals (Ungerstedt, 1971a,b; Zhou and Palmiter, 1995), 1-methyl-4-phenyl-1,2,3,6-tetrahydropyridine (MPTP) poisoning-induced DA loss and motor function loss (Ballard et al., 1985; Langston, 2017), and the dramatic dopaminergic motor stimulation in DA-depleted animals and humans (Cotzias et al., 1969; Ballard et al., 1985; Kim et al., 2000; Li and Zhou, 2013; Lees et al., 2015; LeWitt and Fahn, 2016; Hornykiewicz, 2017).

In both rodents and primates including humans, the striatum receives a very dense DA innervation originated in the substantia nigra (Björklund and Lindvall, 1984; Reiner et al., 1998; Prensa et al., 2000; Lewis et al., 2001; Matsuda et al., 2009; Gerfen and Bolam, 2017; Zhou, 2017). Matching the dense DA innervation, the striatum also expresses a high level of D1 receptors (D1Rs) in the striatal direct pathway medium spiny neurons (dMSNs) projecting to the globus pallidus internal segment (GPi) and substantia nigra pars reticulata (SNr) and also a similarly high level of D2 receptors (D2Rs) in the striatal indirect pathway medium spiny neurons (iMSNs) projecting to globus pallidus external segment (GPe) in rodents and primates including humans (Levey et al., 1993; Yung et al., 1995; Hurd et al., 2001; Gerfen and Bolam, 2017). In addition to the highly concentrated striatal DA system, extrastriatal brain areas also receive DA innervation and express DA receptors. For example, the motor and somatosensory cortical areas in rodents and primates including humans receive DA innervation and express DA receptors, although at lower levels (Gaspar et al., 1991; Smiley et al., 1992, 1994; Smiley and Goldman-Rakic, 1993; Bergson et al., 1995; Ciliax et al., 1995; Yung et al., 1995; Ciliax et al., 1999). Similarly, extrastriatal components of the basal ganglia such as GPe and SNr receive DA innervation and express DA receptors, again at levels much lower than those in the striatum (Cossette et al., 1999; Hedreen, 1999; Prensa et al., 2000; Parent and Cossette, 2001). Furthermore, in PD, besides the severe DA loss in the striatum (Kordower et al., 2013), DA denervation also occurs in GPe and SNr (Hornykiewicz, 1998, 2001; Jan et al., 2000; Rajput et al., 2008), while cortical DA innervation orginating in the ventral tegmental area DA neurons is partially lost (Scatton et al., 1982, 1983; Kish et al., 1988; German et al., 1989; Gaspar et al., 1991; McRitchie et al., 1997; Hornykiewicz, 1998, 2001; Damier et al., 1999; Moore et al., 2008; Kordower et al., 2013; Fu et al., 2016).

In DA replacement therapy for PD, L-dopa and DA agonists are commonly administered orally and thus will activate DA receptors in the striatum and also in extrastriatal brain areas. So an important question is: What are the relative contributions from the DA activity in the striatum and extrastriatal areas to the dopaminergic drugs’ motor-stimulating effects? Further, a previous study suggested that the SNr is the primary site for L-dopa’s motor-stimulating effect in unilateral 6-OHDA rat PD model (Robertson and Robertson, 1989). Thus, a determination of the primary site for the profound motor stimulation will not only advance our understanding about the functional operation of the dMSN- and iMSN-based circuits and their relative importance in dopaminergic motor facilitation, but also provide information for selecting existing dopaminergic drugs for PD patients, developing future anti-PD pharmacotherapies, and for the targeting of potential localized DA replacement therapy for PD.

## Materials and Methods

### Animals

In this study, we used the transcription factor Pitx3 null mutant (Pitx3Null) mice to model the DA denervation in PD (Nunes et al., 2003; van den Munckhof et al., 2003; Ding et al., 2007; Li et al., 2013, 2015; Zhou et al., 2016). These mice have an autogenic, severe and consistent bilateral DA neuron loss in the substantia nigra and DA denervation in the dorsal striatum (DS), whereas about 50% of DA neurons in VTA and hence 50% of DA innervation in the NAc remain (Li et al., 2013; Wei et al., 2013; Ding et al., 2015). Due to the residual DA neurons, these mice survive and reproduce well. Further, the severe DA denervation leads to supersensitive DA receptors in the DS and supersensitive motor response to IP injected L-dopa (Li and Zhou, 2013; Li et al., 2015; Zhou et al., 2016), making Pitx3Null mice a convenient and suitable mouse model to study the consequences of DA loss in PD and to test the motor stimulation of dopaminergic drugs.

Two breeding pairs of heterozygous Pitx3-deficient mice were originally purchased from the Jackson Laboratory (Bar Harbor, ME, United States). A small colony including homozygous Pitx3-/- (Pitx3Null), heterozygous Pitx3+/-, and wild-type Pitx3+/+ (Pitx3WT) mice was generated at the University of Tennessee Health Science Center (UTHSC) (Li and Zhou, 2013; Li et al., 2013; Wei et al., 2013). The genotypes were initially determined by PCR-based genotyping to identify Pitx3WT, Pitx3Null, and heterozygotes (Li et al., 2013). Since Pitx3Null mice are fertile, survive well, and are easily identified by being aphakic (Wei et al., 2013; Zhou et al., 2016), Pitx3Null and Pitx3WT mice for this study were generated by mating Pitx3Null male mice with Pitx3Null female mice (all offspring are Pitx3Null) and mating Pitx3WT male mice with Pitx3WT female mice (all offspring are Pitx3WT) (Wei et al., 2013). Animal care and use were in accordance with federal guidelines and approved by the Institutional Animal Care and Use Committee of UTHSC.

**FIGURE 1:**
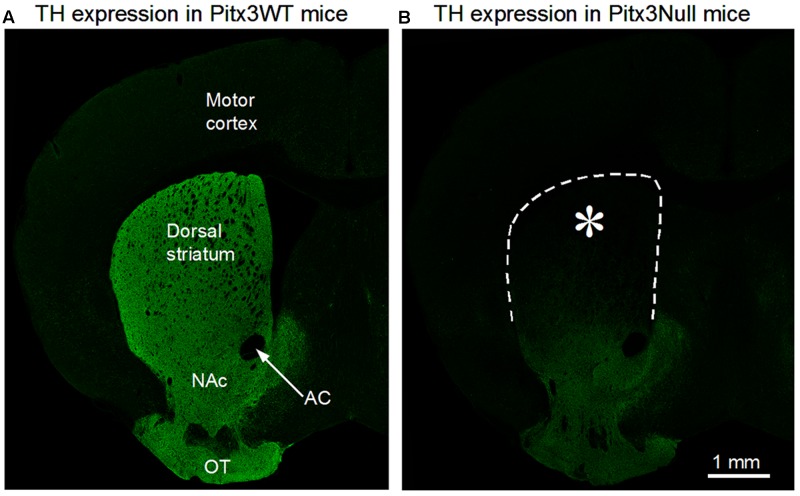
Severe dopamine (DA) denervation in the dorsal striatum (DS) in Pixt3Null mice. **(A)** A confocal microscopic picture of 3 μm optical section showing the normal intense tyrosine hydroxylase (TH) immunostain indicating DA innervation, in the entire striatum in a Pitx3WT mouse. **(B)** A confocal microscopic picture, obtained under identical staining and imaging conditions as in **(A)**, showing the typical gradient pattern of DA denervation in the striatum in Pixt3Null mice. Note that the DS is virtually void of TH stain or DA axons, whereas the ventral striatum including the nucleus accumbens (NAc) retains substantial amounts of DA innervation. DA innervation is bilateral and symmetrical but only one hemisphere is shown. AC, anterior commissure; OT, olfactory tubercle. ^∗^Indicates the microinjection target area in the DS.

We and others have documented the gradient striatal DA axon loss in Pitx3Null mice (Nunes et al., 2003; van den Munckhof et al., 2003; Ding et al., 2007, 2015; Li and Zhou, 2013; Wei et al., 2013). To provide a firm foundation for our present study and also to help the reader to understand the data and interpretation of this present study, here we replicated the published gradient DA denervation in the striatum in Pitx3Null mice (**Figure 1**). A key advantage of Pitx3Null mice is that its striatal DA denervation is autogenic and consistent among every Pitx3Null mouse, and the DA denervation pattern resembles the DA denervation pattern in PD. Although the DA loss in Pitx3Null mice is not progressive, unlike that in PD, but the DA denervation-induced molecular, cellular and behavioral DA supersensitivity appears to be similar or identical in Pitx3Null mice, other animal PD models and human PD brains (Pifl et al., 1992; Corvol et al., 2004; Ding et al., 2007, 2015; Li and Zhou, 2013; Wei et al., 2013; Li et al., 2015). Thus, Pitx3Null mice are suitable for studying the consequences of DA denervation, matching the goal of our present study.

We used 4–5 months old male Pitx3Null mice in this study. The mice were housed in groups of five before implantation surgery and individually after surgery in a temperature and humidity-controlled room within the UTHSC animal facility. They had free access to food and water. Mice were divided randomly into four groups according to different brain regions: groups of primary motor cortex (MC), DS, SNr, GPe. The procedures were approved by UTHSC Institutional Animal Care and Use Committee.

### Immunohistochemistry

Conventional immunofluorescence methods were used to detect DA axons in the striatum (Zhou et al., 2009; Li and Zhou, 2013; Wei et al., 2013; Ding et al., 2015). The brains were fixed in 4% paraformaldehyde (PFA) dissolved in phosphate buffered saline (PBS) at 4°C overnight and then sectioned on a vibratome. The free-floating sections (50 μm in thickness) were incubated with 2% fat-free milk, 1% bovine serum albumin, and 0.4% Triton X-100 in PBS for 1 h at room temperature to block non-specific binding and permeabilize the cell membrane, respectively. After thorough rinsing, the free-floating sections were incubated for 48 h at 4°C with the primary antibody, a polyclonal tyrosine hydroxylase (TH) antibody raised in rabbit (diluted at 1:1000; Novus Biologicals, Littleton, CO, United States), and then rinsed in PBS, followed by incubating with a donkey anti-rabbit secondary antibody conjugated with the green Alexa Fluor 488 (diluted at 1:200; Invitrogen), for 3 h at room temperature. Confocal microscopic images were acquired on a Zeiss 710 laser scanning confocal microscope.

### Stereotaxic Surgery

Mice were anesthetized with ketamine and xylazine (100 and 10 mg/kg, respectively, i.p.) and placed in a Kopf digital motorized stereotaxic apparatus. Following the standard antiseptic procedure practiced in the lab (Li et al., 2015) and the mouse brain atlas of Paxinos and Franklin (2001), 26 gauge microinjection guide cannulas (Plastics One, Roanoke, VA, United States) were implanted bilaterally (bilateral implantation was both for drug infusion and for controlling for any potential behavioral consequences of the implanted cannula). To reduce tissue damage, the tip of the 26 gauge guide cannula was 0.5–1.0 mm above the target location, and the much smaller (33 gauge) internal injection cannula were designed to reach the target with much smaller tissue damage. The coordinates are the following. For MC, it was AP 0.74 mm; ML ± 1.5 mm from bregma and DV -1.5 mm from the surface of skull); for DS, it was AP 0.3 mm, ML ± 2 mm from bregma and DV -3.0 mm from the surface of skull); for SNr, it was AP -3.16 mm, ML ± 1.5 mm from bregma and DV -4.25 mm from the surface of skull; and for GPe, it was AP -0.46 mm, ML ± 2 mm from bregma and DV -3.75 mm from the surface of skull. The implanted guide cannulas were fixed onto the skull by dental cement and four stainless steel screws anchoring into the skull. Dummy internal cannulas (i.e., protective plugs) were inserted into guide cannulas to prevent occlusion. Immediately after surgery, the mouse was warmed by a heating pad and treated with the analgesic buprenorphine at 0.1 mg/kg to relieve the likely pain from the surgery. The mice were also provided moist food pellets in the first week after surgery and 2 weeks were given for recovery from the surgery.

### Drugs

Four different drug solutions were prepared in this experiment: L-dopa, D1-like receptor agonist SKF81297 (Andersen and Jansen, 1990), D2-like receptor agonist ropinirole (Millan et al., 2002, 2004) and a mixture of SKF81297 and ropinirole. All the drugs were dissolved in sterile saline (0.9%), aliquoted, and stored in -20°C. The dose was 1 μg for L-dopa, 0.2 μg for SKF81297 and ropinirole, all in 0.2 μL saline. For mixed SKF81297 and ropinirole injection, 0.2 μg SKF81297 and 0.2 μg ropinirole were dissolved in 0.2 μL saline. Ropinirole and SKF81297 were purchased from Tocris; L-dopa (L-3,4-Dihydroxyphenylalanine methyl ester hydrochloride) was purchased from Sigma.

### Intracranial Drug Microinjection

Two weeks after implanting the guide cannulas, intracranial drug microinjection was performed using a 10 μL Hamilton microsyringe driven by a Harvard Apparatus syringe pump. The microsyringe needle was connected to a 70 cm long polyethylene (PE)-10 tubing that was in turn connected to the 33 gauge internal cannula. The injection system was first tested for leaks by filling the microsyringe and tubing with distilled water. Then all the water in microsyringe was expelled out through the internal injection cannula to make sure that there was no leak in the system. Finally, the drug solution was drawn into the PE-10 tubing via internal cannula after 2–3 cm pocket of air was pulled into the tubing to facilitate the visual monitoring of the movement of the small volume, colorless drug solution in the tubing, and the injection system was readied by observing a drop of drug solution at the tip of the connected internal injection cannula.

After the mouse was hand-restrained by the experimenter, the dummy cannula was removed and the internal injection cannula, with a drop of drug solution at the tip, was inserted into the guide cannula and then into one of the four target brain areas (MC, DS, GPe, and SNr). Then the syringe pump was activated and the drug solution was injected into the target brain area at a flow rate of 40 nL/min, and the injection of 0.2 μL drug solution lasted 5 min. The internal cannula was kept in the brain for additional 5 min to allow the drug to diffuse into the surrounding tissue and also to prevent the drug solution to flow into the needle-produced tissue cavity. Then the internal cannula was removed gently and the syringe pump was restarted for observing drops of drug solution coming out from the internal cannula to re-confirm that the injection system was working and the injection cannula and the tubing were not clogged or leaking. For each mouse, only one brain area was targeted bilaterally, and each brain area was microinjected with only one drug.

### Behavioral Assessment

Behavioral test was carried out with a 24 cm × 24 cm square recording cage and a video-recording camera after at least 2 weeks recovery from cannula implantation surgery. For the behavioral test, a mouse was gently placed into the recording cage and acclimated to this situation for more than 60 min. When the mouse calmed down, intracranial drug microinjection was accomplished as described above. The behavioral recording started 60 min before intracranial microinjection and lasted up to 2 h after microinjection to record the full course of the behavioral effects of the injected drug. The rotations induced by injected drug were counted manually and averaged every 10 min during the full course of recording.

Unilateral drug infusion-induced asymmetrical, one-sided movement (rotation) is a sensitive readout of the drug’s motor stimulation (Lane et al., 2006). Additionally, rotations can be recognized and counted reliably and easily (Ungerstedt, 1971a; Schwarting and Huston, 1996; Lane et al., 2006).

In each behavioral test, only one drug was injected into one brain area. Since symmetrical bilateral injection is often not achieved due to inherent difficulties and variation in microinjection of 0.2 μL into tissue, unilateral injection was used in this study and the consequent rotational movement was used as the readout of motor activity, taking the advantage that rotations are easily identified and also a commonly used parameter.

To control for any potential non-specific effect of the injection procedure, saline (0.2 μL) was microinjected into DS, GPe, SNr and MC, and no rotation was induced (*n* = 2–3 mice for each site).

### Histology

To mark the internal cannula track, the internal cannula was left in the guide cannula and target brain area for 24 h after the completion of experiments. Then mice were overdosed with ketamine and xylazine, and intracardiacally perfused with PBS followed by 4% PFA. Then the brain was dissected out and post-fixed in 4% PFA overnight. Coronal sections (0.1 mm thick) were cut on a vibratome and observed and photographed under an Olympus microscope to verify the locations of the guide cannula and internal injection cannula. Data from mice with misplaced cannulas were excluded from analysis.

### Statistics

Numerical values were presented as the Mean ± SEM and analyzed by Graphpad Prism 5. The difference between the numbers of rotation before and after microinjection of a drug was compared by using two-way analysis of variance (two-way ANOVA) with one repeated measure (time). Two-way ANOVA was also used to compare the numbers of rotation induced by different drugs. The total numbers of rotation induced by the same drug in MC, SNr, and GPe were compared with that in DS by one-way ANOVA followed by Tukey’s Multiple Comparison Test. *p* < 0.05 was considered to be statistically different.

## Results

### Unilateral L-Dopa Microinjection into DS, SNr, GPe, and MC to Determine the Primary Site for L-Dopa to Stimulate Motor Activity

Since our goal is to compare the relative contribution of the striatal DA system and extrastriatal DA system to the motor-stimulating effect of the DA replacement therapy in a PD mouse model, the Pitx3Null mice, we set out to first determine and compare the motor-stimulating effect of L-dopa in the DS, GPe, SNr, and MC. Due to DA denervation-induced DA receptor supersensitivity, we used the unilateral drug injection-induced contralateral rotation as a reliable, easy-to-recognize and easy-to-quantify readout of dopaminergic motor stimulation.

**FIGURE 2:**
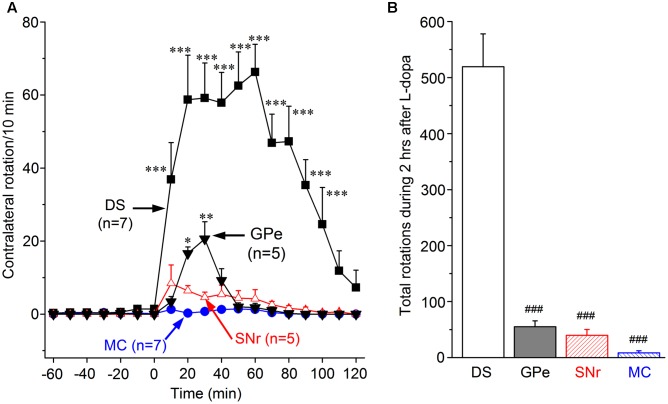
Motor-stimulating effects of L-dopa unilateral microinjection into the DS, globus pallidus external segment (GPe), substantia nigra pars reticulata (SNr), and primary motor cortex (MC). **(A)** Contralateral rotations triggered by 1 μg L-dopa (in 0.2 μL saline) microinjection in DS, GPe, SNr, and MC. ^∗^*p* < 0.05, ^∗∗^*p* < 0.01, ^∗∗∗^*p* < 0.001 compared with the baseline. **(B)** Cumulative total number of contralateral rotations during the 2 h after 1 μg L-dopa microinjection into DS, GPe, SNr, and MC. ^###^*p* < 0.001 compared with the DS group.

We used digital video cameras to record the motor behavior of the mice, and the digital videos were viewed and analyzed off-line. The baseline recording started 1 h before drug microinjection and lasted 2 h after drug microinjection. As shown in **Figure 2A**, no asymmetrical movement or rotation was detected during the baseline recording. Upon microinjection of 1 μg L-dopa dissolved in 0.2 μL saline into the DS, the mice started to show asymmetrical, contralateral rotation about 4 min after the start of the microinjection, before the withdraw of the injection needle at the end of 5th min. Ten min after the start of the L-dopa microinjection into DS, rotational movements were robust; this effect reached its peak (58.7 rotations/10 min) at 20 min after injection, plateaued for 40 min, then slowly declined, and ended around 110 min after the start of the L-dopa microinjection (**Figure 2A**).

We then repeated the experiment in GPe and SNr, two extrastriatal brain areas that lose substantial amounts of DA innervation in PD brains (Hornykiewicz, 2001) and Pitx3Null mice (Wei et al., 2013). We found that L-dopa (1 μg in 0.2 μL) microinjection into GPe triggered asymmetric rotations, although this effect was modest and much weaker than the effect induced by L-dopa (**Figures 2A,B**). We also found that L-dopa (1 μg in 0.2 μL) microinjection into SNr triggered only a minimal number of contralateral rotations. Finally, we microinjected 1 μg L-dopa into the MC and detected no rotation or other movement during the 2 h after L-dopa injection (**Figures 2A,B**).

The cumulative total numbers of rotation in 2 h after L-dopa microinjection in the four brain areas are shown in **Figure 2B**. It is clear that the number of rotation induced by L-dopa in DS (519.4 ± 58.6) is far more than those induced by L-dopa in GPe (55.0 ± 10.4), SNr (39.8 ± 10.4), and MC (8.3 ± 3.7).

These data show unambiguously that L-dopa microinjection into the DS induced the strongest motor activity, whereas L-dopa microinjection into the GPe, SNr, and MC induced only very weak or minimal motor activity.

### Unilateral D1-Like Agonist SKF81297 Microinjection into DS, SNr, GPe, and MC to Determine D1R Agonism’s Stimulation of Motor Activity and Its Primary Site of Action

To further determine the relative contribution of the striatal and extrastriatal DA system to the dopaminergic stimulation of motor activity, and to exclude the possibility that the weak motor-stimulation of L-dopa injection into GPe, SNr, and MC may be due to a failure to convert sufficient amounts of L-dopa to DA, we tested direct dopaminergic agonists. Here we first describe the results from microinjecting SKF81297, a full agonist for D1Rs and also D5Rs (Andersen and Jansen, 1990). Results on the effects of a D2R-like agonist are described in Section “Unilateral D2-Like Agonist Ropinirole Microinjection into DS, SNr, GPe, and MC to Determine D2R Agonism’s Stimulation of Motor Activity and its Primary Site of Action” below.

**FIGURE 3:**
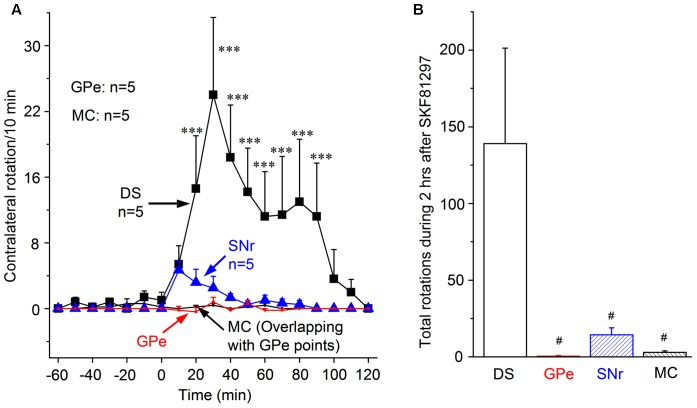
Contralateral rotations induced by SKF81297 unilateral microinjection into DS, GPe, SNr, and MC. **(A)**. Contralateral rotations triggered by 0.2 μg SKF81297 (in 0.2 μL saline) microinjection in DS, GPe, SNr, and MC. ^∗∗∗^*p* < 0.001 compared with the baseline. Note there was practically no contralateral rotations triggered by SKF81297 microinjection in GPe and MC such that the two lines are at the zero level and hence overlap. **(B)** Cumulative total numbers of contralateral rotations during the 2 h after 0.2 μg SKF81297 microinjection into DS, GPe, SNr, and MC. ^#^*p* < 0.05 compared with the DS group.

As shown in **Figure 3A**, microinjection of 0.2 μg SKF81297 (in 0.2 μL saline) into the DS induced moderate numbers of contralateral rotation. The effect was clear at 20 min after microinjection. The peak effect, reached at 30 min after microinjection, was 26.0 ± 9.4 rotations/10 min. The effect then gradually declined, and disappeared at 90 min. The cumulative total contralateral rotation number during the 2 h following microinjection was 139.0 ± 62.3 (**Figure 3B**). By comparing with data in **Figure 2**, it is amply clear that this SKF81297-triggered response was much weaker than that triggered by L-dopa (see **Figure 2**). This difference is not due to insufficient amounts of SKF 81297 but due to L-dopa’s concurrent activation of D1Rs and D2Rs, as documented below in Section “Unilateral Concurrent Microinjection of SKF81297 and Ropinirole into the DS to Determine the Potential Synergistic Effects of D1R and D2R Agonism.”

SKF81297 (0.2 μg in 0.2 μL saline) microinjection into SNr also produced only a small number of rotations, and little to no effect was detected after SKF81297 microinjection into GPe or MC (**Figures 3A,B**). The cumulative total number of rotation induced by SKF81297 microinjection in DS, GPe, SNr, MC are 139.0 ± 62.3, 0.4 ± 0.5, 14.33 ± 4.6, 3.0 ± 0.9, respectively (**Figure 3B**). The number of rotation induced by SKF81297 in DS is clearly more than those in SNr, GPe, and MC. This is probably because striatonigral neurons in the striatum express the highest amount of D1Rs and these D1Rs are critical to the spike firing of striatonigral neurons; the striatonigral axon terminals in SNr also express substantial amounts of D1Rs, whereas GPe is not known to express D1Rs, and MC has only low levels of D1R expression. If GPe neurons express D1Rs/D5Rs, the effect should be inhibiting and impairing motor activity. The results above demonstrate that D1R agonist SKF81297 in DS (stimulating the somatic D1Rs) is the most effective means (likely to help triggering action potentials) for D1R agonism to stimulate motor activity, whereas stimulating axon terminal D1Rs is much less effective.

### Unilateral D2-Like Agonist Ropinirole Microinjection into DS, SNr, GPe, and MC to Determine D2R Agonism’s Stimulation of Motor Activity and Its Primary Site of Action

For this experiment, we chose ropinirole to activate striatal D2Rs because it is a currently used frontline drug for PD (Katzenschlager et al., 2008; Olanow et al., 2009; Connolly and Lang, 2014). Its low affinity to D2Rs (and D4R) (both *K*i = 800 nM) and high affinity to D3Rs (*K*i = 37 nM) (Millan et al., 2002, 2004) do not affect the interpretation of our results–this is further addressed in Section “Discussion.” Ropinirole can also activate D2Rs expressed on the striatopallidal axon terminals (Wei et al., 2013, 2017).

**FIGURE 4:**
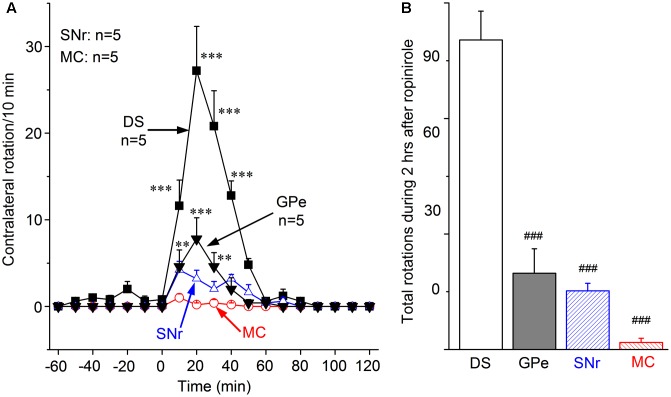
Motor-stimulating effect of ropinirole unilateral microinjection into DS, GPe, SNr, and MC. **(A)** Contralateral rotations triggered by 0.2 μg ropinirole (in 0.2 μL saline) microinjection in DS, GPe, SNr, and MC. ^∗∗^*p* < 0.01, ^∗∗∗^*p* < 0.001 compared with the baseline. Note there were practically no contralateral rotations triggered by ropinirole microinjection in MC. **(B)** The total number of contralateral rotation induced by ropinirole microinjection in DS, GPe, SNr, MC. ^###^*p* < 0.001 compared with DS group.

As illustrated in **Figure 4A**, 0.2 μg ropinirole (in 0.2 μL saline) microinjection into the DS triggered moderate numbers of contralateral rotation. This effect was clear at 10 min after the microinjection, reached the peak of 27.2 rotations/10 min at 20 min after injection, then declined, and disappeared at 50 min after microinjection. When compared with its baseline, the number of rotation after DS ropinirole microinjection increased significantly from 10 min through 40 min after microinjection.

In addition, ropinirole microinjection into GPe produced a small but significant number of rotations (**Figures 4A,B**). Ropinirole microinjection into SNr produced a further smaller number of rotations, and practically no rotation was triggered when ropinirole was microinjected in the MC (**Figures 4A,B**). During the 2 h following ropinirole microinjection in DS, GPe, SNr and MC, the cumulative total number of rotation is 80.4 ± 7.5, 19.8 ± 6.4, 15.2 ± 2.0, and 1.8 ± 1.1, respectively. These results clearly indicate that the DS is the primary site for ropinirole to stimulate motor function.

### Unilateral Concurrent Microinjection of SKF81297 and Ropinirole into the DS to Determine the Potential Synergistic Effects of D1R and D2R Agonism

The much stronger motor-stimulating effect produced by L-dopa microinjection into the DS than that produced by SKF81297 or ropinirole (**Figures 2–4**) suggests that concurrent activation of D1Rs in dMSNs and D2Rs in iMSNs may function synergistically to produce stronger motor behavioral outputs. But comparison of the effects of L-dopa and SKF81297 and ropinirole is confounded by the differences in their affinity to DA receptors and other aspects of these drugs. To avoid these confounding factors, we have tested the motor effects of concurrent microinjection of SKF81297 and ropinirole (0.2 μg each in a total of 0.2 μL saline, i.e., a mix of these two drugs) into DS.

**FIGURE 5:**
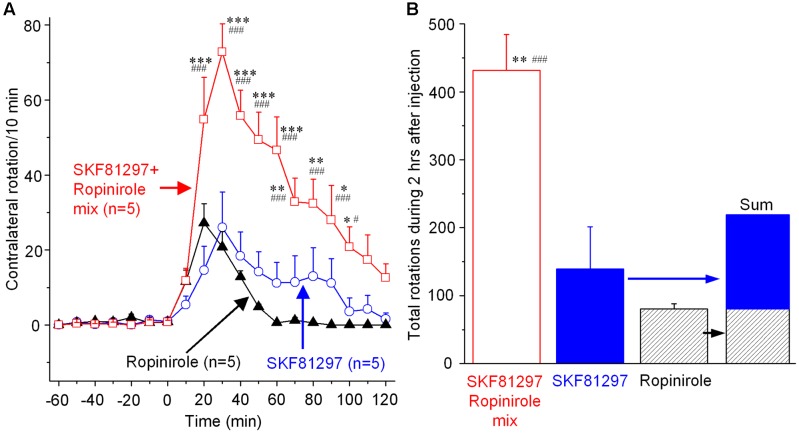
Contralateral rotations induced by concurrent SKF81297 and ropinirole unilateral microinjection into the DS. **(A)** Contralateral rotations triggered by unilateral DS microinjection of 0.2 μg SKF81297-0.2 μg ropinirole mix (in 0.2 μL saline), 0.2 μg SKF81297 alone (in 0.2 μL), and 0.2 μg ropinirole alone (in 0.2 μL). ^∗^*p* < 0.05, ^∗∗^*p* < 0.01, ^∗∗∗^*p* < 0.001 compared with SKF81297 group. ^#^*p* < 0.05, ^###^*p* < 0.001 compared with ropinirole group. **(B)** Cumulative total numbers of contralateral rotations during the 2 h after the unilateral DS microinjection of SKF81297-ropinirole mix, SKF81297 alone and ropinirole alone. ^∗∗^*p* < 0.01 compared with SKF81297 group, ^###^*p* < 0.001 compared with ropinirole group.

As shown in **Figure 5**, microinjection of the SKF81297-ropinirole mix into the DS triggered robust contralateral rotations. The effect reached its peak of 72.8 rotations/10 min at 30 min after the microinjection. Then the effect declined slowly and disappeared at 2 h after the microinjection (**Figure 5A**). This effect is much stronger than the effect induced by microinjection of SKF81297 or ropinirole alone, as shown in **Figures 3**, **4**. To better illustrate the difference, the individual effects of SKF81297 and ropinirole microinjection into DS are re-plotted here in **Figures 5A,B**. The combined data show unambiguously that the number of contralateral rotations produced by the SKF81297 and ropinirole mix was larger than the sum of the rotations produced by separate microinjections of SKF81297 and ropinirole. Specifically, the cumulative total number of rotation induced by the SKF81297-ropinirole in the 2 h was 431.6 ± 53.1, whereas the number of SKF81297-induced rotation was only 139.0 ± 62.3, and the number of ropinirole-induced rotation was only 80.4 ± 7.5. This result clearly indicates a synergy between D1R activation in dMSNs and D2R activation in iMSNs.

### Anatomical Verification of Cannula Placement in the Four Target Brain Areas

**FIGURE 6:**
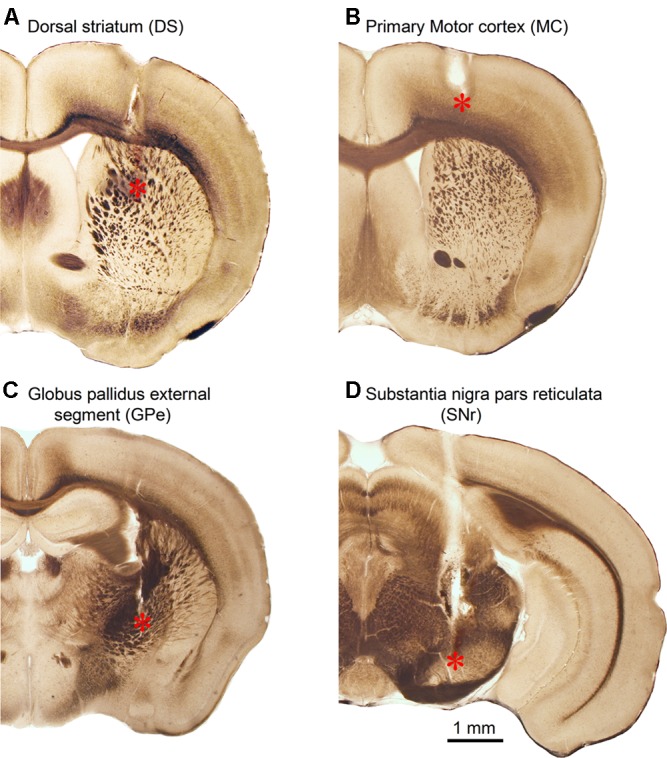
Anatomical verification of internal injection cannula tip location in the DS **(A)**, primary MC **(B)**, GPe **(C),** and SNr **(D)**. ^∗^Indicates internal injection cannula tip location.

After completing drug microinjection and behavioral experiments, mice were euthanized and the brains were dissected out, fixed, sectioned, and examined under a microscope (see section “Materials and Methods”). **Figure 6** shows representative photomicrographs of guide cannula and internal cannula tracks in the DS, GPe, SNr, and MC. The chronically implanted guide cannula track was relatively large and clear, whereas the transiently inserted internal injection cannula track was relatively small but still visible (**Figure 6**). This difference is expected because for reducing cannula-induced tissue damage in the target area, we chronically implanted the relatively large 26 gauge guide cannula in a way such that the tip was not in the target area and we inserted the smaller 33 gauge internal injection cannula only transiently for injection. Further, to avoid tissue section breakage and loss during staining processes, these tissue sections were not stained, but these tissue sections still show the cannula tracks. Additionally, histochemical staining for DA is not needed here, because DA neuron loss and DA denervation in Pitx3Null mice are well established by the work from several laboratories including our own work (Nunes et al., 2003; van den Munckhof et al., 2003; Ding et al., 2007, 2015; Li and Zhou, 2013; Wei et al., 2013), and this DA denervation is identical in every Pitx3Null mouse, unlike toxin-induced DA denervation that is variable and needs to be determined in each mouse.

## Discussion

Our main findings are that (1) microinjection of L-dopa, D1R agonist SKF81297 or D2R agonist ropinirole into the DS each induced strong motor activity with L-dopa’s effect being the strongest; the same drugs microinjected into GPe, SNr, or MC induced weak motor activity, indicating the DS is the primary site for dopaminergic motor stimulation; and (2) compared with SKF81297 or ropinirole microinjection, L-dopa or concurrent SKF81297 and ropinirole microinjection into the DS stimulated motor activity more strongly than the sum of the motor stimulation induced by separate microinjection of SKF81297 and ropinirole, indicating a synergy between dMSN-based circuit and iMSN-based circuit in facilitating motor function.

### The Striatum, Not GPe, SNr, or Motor Cortex, Is the Primary Site for L-Dopa and DA Agonists to Stimulate Motor Activity

We found that dorsal striatal microinjection of L-dopa, D1R agonist SKF81297 and D2R agonist ropinirole each induced robust motor responses; in contrast, microinjection of these same drugs into GPe, SNr, or MC induced only weak or minimal motor stimulation. These results indicate that striatal but not extrastriatal DA receptors are critical to DA’s profound motor stimulation in parkinsonian animals and PD patients.

The very weak motor-stimulating effect of L-dopa or SKF81297 and ropinirole in the MC is likely due to the fact that in both rodents (Mansour et al., 1990, 1991, 1992a; Meador-Woodruff and Mansour, 1991; Levey et al., 1993) and primates including humans (Mansour et al., 1992b; Meador-Woodruff et al., 1993; Hurd et al., 2001), DA receptor expression in the MC is at a low level, much lower than that in the striatum, also lower than that in GPe and SNr. Another reason for the weak motor-stimulating effect of L-dopa or SKF81297 and ropinirole in the MC may be that for already learned, automated movements such as locomotion (rotation is likely a form of unbalanced locomotion), the MC may be not needed and the brainstem and the striatum are more important (Roseberry et al., 2016; Takakusaki, 2017). Thus, dopaminergic activity in the MC does not affect locomotion, whereas increased dMSN output may, via basal ganglia output nuclei, stimulate the brainstem and spinal cord locomotion center and increase locomotion, without involving the MC (Grillner et al., 2005).

The much lower motor-stimulating effects of L-dopa and D1R and D2R agonists in GPe and SNr than those in the DS can be easily understood based on the intense DA innervation and D1R and D2R expression that are far higher than those in the GPe and SNr in both rodents and primates including humans (Mansour et al., 1992b; Meador-Woodruff et al., 1993; Hurd et al., 2001). Taken together, our results clearly indicate that the heavy DA receptor expression in the striatum is directly related to the powerful motor-stimulating function of the striatum and striatum-microinjected dopaminergic drugs. Thus, the striatum (dMSNs and iMSNs and their D1Rs and D2Rs) is the primary target of the DA replacement therapy for PD, and extrastriatal brain areas are probably not suitable targets for localized or precision DA replacement therapy for PD.

Extrastriatal DA systems are being increasingly recognized with better anatomical detection and may contribute to the normal brain physiology and also disease pathophysiology (Smith and Villalba, 2008; Rommelfanger and Wichmann, 2010; Benazzouz et al., 2014), but their functional roles in motor behavior and dopaminergic motor stimulation in PD are not established. Our present study suggests that extrastriatal DA systems play only a minimal role in the profound dopaminergic motor stimulation; the striatal DA system is the main mediator.

Ropinirole has a relatively low affinity to D2Rs and D4Rs (*K*i ∼ 800 nM) and a higher affinity to D3Rs (*K*i ∼ 37 nM) (Millan et al., 2002, 2004). We chose ropinirole in this study for its clinical importance because it is a currently used frontline drug for PD (Katzenschlager et al., 2008; Olanow et al., 2009; Connolly and Lang, 2014). We argue that under our experimental condition, ropinirole exerted its motor-stimulating effect primarily by activating D2Rs for the following reasons. First, in the DS, D3R expression is very low (Lévesque et al., 1992), D4R expression is also very low (Mauger et al., 1998), whereas D2R expression is overwhelmingly high (Levey et al., 1993). Thus, D2R-mediated effects will be the predominant. Second, genetic deletion of D3Rs and D4Rs each causes little impairment on motor function (Accili et al., 1996; Rubinstein et al., 1997; Thanos et al., 2010). In fact, D3R KO leads to motor hyperactivity (Accili et al., 1996), indicating D3Rs may be motor-inhibitory, potentially via mechanisms in brain areas not examined in this study; whereas D2R deletion leads to severe motor deficits as fully expected based on the classic BG model (Baik et al., 1995; Bello et al., 2017). Third, we have determined that the D3 antagonist GR 103691 does not affect L-dopa-induced motor activity in Pitx3Null mice, indicating that D3Rs do not make a detectable contribution to dopaminergic motor stimulation in our mouse model. Fourth, we microinjected 200 ng of ropinirole in 0.2 μL (0.2 mm^3^) saline, i.e., the ropinirole concentration at the needle tip is 3.85 mM. Let’s assume ropinirole diffuses 1 mm in all direction from the needle tip in the DS center [the lateral-medial width of the DS (one side) is only about 2 mm in mice], this leads to a sphere 4.2 mm^3^ and a 20-fold dilution, producing an average ropinirole concentration of 190 μM, still a saturating concentration at D2Rs. We have reason to believe that the drug diffusion distance was less than 1 mm and even less than 0.5 mm based on the fact that injection of L-dopa or other drugs into the deep layers of the MC induced virtually no motor stimulation, even though this injection site is only about 0.5 mm above the DS where DA receptors are supersensitive (Ding et al., 2015) and L-dopa induced strong rotation (**Figure 2**); if these drugs can diffuse 0.5–1.0 mm, then their injection into this area would have induced clear motor stimulation. Thus, taken together, our microinjected ropinirole in the DS likely exerted its motor-stimulating effects primarily through D2Rs in iMSNs.

Our results are consistent with the anatomical arrangement that the striatum has a much more intense DA innervation than GPe and SNr, i.e., the striatum, not GPe or SNr, is built to be the brain structure for intense DA signaling that facilitates cortical motor commands and consequently stimulates motor function. Certainly, DA activity in the extrastriatal brain areas can contribute to motor function, but this contribution is small compared with the DA system in the striatum. Our results are also consistent with the findings of Singh et al. (2016) that the striatum is the primary site of DA denervation-induced pathophysiology that can lead to secondary pathophysiological changes in extrastriatal brain areas.

Our data show that DA’s somatic effects in medium spiny neurons (MSNs) are more important to motor function because the somatic effect affects the generation of action potentials that trigger GABA release, whereas axon terminal D1Rs or D2Rs can only enhance or damp this release process. This is totally reasonable because triggering or inhibiting somatic action potentials that in turn trigger GABA release is obviously more important than the axon terminal effects that enhance or damp action potential-triggered GABA release.

### Synergistic Motor-Stimulating Effect of Concurrent D1 and D2 Agonism in the Striatum

Our data show that microinjection of a mix of D1 agonist SKF81297 and D2 agonist ropinirole into the DS produced a motor stimulation that was larger than the sum of the motor stimulation produced by separate SKF81297 and ropinirole microinjections. Thus, our data show that D1R agonism and D2R agonism each triggers movements but a combined D1R and D2R agonism (L-dopa or a mix of D1 agonist SKF81297 + D2 agonist ropinirole) is more effective, indicating individual contributions from dMSNs and iMSNs and also a dMSN-iMSN synergy in triggering movements. Our results are consistent with the finding that in the DS, dMSNs and iMSNs function in a complementary manner to produce coordinated motor behavior (Cui et al., 2013; Friend and Kravitz, 2014; Tecuapetla et al., 2016). It is likely that D1R agonism and hence the cAMP-producing D1R activation will increase the activity of the motor-promoting dMSN activity, and D2R agonism and hence cAMP-lowering D2R activation will decrease the motor-inhibiting iMSN activity. Thus, the DA system in the striatum, via the concurrent activation of the intensely expressed D1Rs in dMSNs and equally intensely expressed D2Rs in iMSNs highly efficiently facilitates motor functions. These findings not only advance our understanding about the functional operation of the striatal circuitry, but also have therapeutic implications for PD, that is, agents with mixed D1R and D2R agonistic properties are better than pure D1R or D2R agonists. The excellent therapeutic efficacy of L-dopa (converted to DA in the brain) for PD is consistent with our idea. But L-dopa becomes less effective in late stage PD as most DA terminals are lost such that L-dopa cannot be efficiently converted to DA and stored in DA terminals for later release. Thus, direct DA receptor agonists are useful. Currently used ropinirole and pramipexole each activates D2Rs (and also D3Rs and D4Rs) only. We suggest that direct DA receptor agonists activating both D1Rs and D2Rs will have better therapeutic effects. We also need to note here that this D1R–D2R synergy is not likely due to D1Rs and D2Rs co-expressing in the same MSNs because D1Rs and D2Rs are practically completely segregated in the dMSNs and iMSNs (Valjent et al., 2009; Gerfen and Surmeier, 2011). Hence, this synergy is due to collaborative interaction of the D1R-expressing dMSNs-based circuitry and the D2R-expressing iMSNs-based circuitry in the motor control system.

Behavioral synergism between D1R-like agonism and D2R-like agonism has been reported (Paul et al., 1992; Robertson, 1992; LaHoste et al., 1993, 2000; Capper-Loup et al., 2002), but these studies used systemic drug administration, creating an uncertainty about the precise site of the drug action. Our present study employed the microinjection technique and thus avoids the uncertainty of these prior studies. Our results advance the research field by showing that the striatal D1Rs and D2Rs are the primary mediators of D1R–D2R synergism.

## Conclusion

Our data indicate that striatal D1Rs and D2Rs in the striatal direct and indirect pathway spiny projection neurons, not in neurons in extrastriatal brain areas, are the primary targets for the DA replacement therapy for PD. Our data also suggest that D1Rs and D2Rs in the striatum-based circuits work synergistically to facilitate motor function such that L-dopa stimulates motor activity more strongly than a selective D1R or D2R agonist, and hence broad spectrum DA agonists are likely to be more effective anti-PD drugs than the current D2-like receptor DA agonist drugs.

## Author Contributions

YW and F-MZ: contributed in project conception and design, data collection, analysis and interpretation, and manuscript writing.

## Conflict of Interest Statement

The authors declare that the research was conducted in the absence of any commercial or financial relationships that could be construed as a potential conflict of interest.
